# Supplementary material for: Cerebral cortical structures linked to intelligence
Source: bioRxiv. 2025 Oct 7:2025.10.06.680808. Preprint. [Version 1] doi: 10.1101/2025.10.06.680808 (PMC12632532; doi:10.1101/2025.10.06.680808)
Supplement: Supplement 1 [file media-1.pdf]

Supplementary Materials:

Cerebral cortical structures linked to intelligence

Table of Contents

Supplementary Methods.
Supplementary Results.
SFigure 1.
Supplementary Table.
Supplementary Reference.

Supplementary Methods.

1. Details of Mendelian Randomization Analyses

To evaluate the robustness of our causal inference and address potential violations of MR assumptions, we implemented five complementary MR approaches: GSMR (1), inverse-variance weighted (IVW), MR-Egger regression, weighted median, and weighted mode (2–4). Each method relies on different assumptions and offers different sensitivity to pleiotropy or invalid instruments.

For GSMR, SNP instruments were first selected from the exposure GWAS at genome-wide significance (p < 5e-8) and pruned for linkage disequilibrium ($r^2 < 0.05$, 250kb). For each retained SNP, we calculated instrument strength using the F-statistic, with all instruments exceeding the conventional threshold of $F > 10$, ensuring sufficient strength. While Mendelian Randomization guidelines suggest a rule of thumb of at least 10 independent instruments for robust sensitivity analyses, prior work has emphasized that analyses with fewer variants can still yield valid inference if the instruments are strong and results are interpreted cautiously (5, 6). This applied to our reverse MR models, where fewer than 10 instruments were available but all exhibited strong F-statistics. GSMR applies the HEIDI-outlier test (pHEIDI < 0.01) to identify and remove individual SNPs whose outcome effects deviate from expectation, thereby filtering potential pleiotropic variants before causal estimation. While this approach directly addresses exclusion restriction violations, residual pleiotropy may persist if effects are small or widespread. IVW provides high statistical power by combining all instruments under the assumption that they are valid or that pleiotropic effects are balanced on average; however, it is vulnerable to directional pleiotropy. MR-Egger regression relaxes this assumption by allowing for a non-zero intercept, which can detect and adjust for directional pleiotropy, but requires the InSIDE assumption and has lower power with wider confidence intervals. The weighted median estimator yields consistent causal effects if at least 50% of the total instrument weight comes from valid SNPs, offering robustness to invalid instruments at the cost of some efficiency. Finally, the weighted mode method identifies the most common causal effect across instruments and assumes that the largest cluster represents valid instruments, providing robustness in cases where only a subset of SNPs are valid but with reduced statistical power. By aligning the results across these complementary methods, we were able to assess the stability of causal estimates under different model assumptions and better evaluate the potential impact of assumption violations.

2. Details of GSMR exposure and outcome

For the exposures, we used genome-wide association results of cortical morphology derived from MRI-based measures of cortical surface area and thickness in

the UK Biobank imaging cohort (N ≈ 33,000 after quality control) (7, 8). For the outcome, we used publicly available summary statistics from the large-scale genome-wide association study of intelligence reported by Savage et al (9). This meta-analysis included approximately 270,000 individuals of European ancestry from multiple cohorts (e.g., UKB, COGENT, RS, STR, TEDS). Intelligence was measured through harmonized cognitive performance assessments across contributing studies. Different cognitive measures were harmonized to index a common latent *g* factor. To avoid sample overlap with our UKB brain morphology GWAS, we specifically requested and used the UKB-excluded summary statistics provided by the authors, which were based on N = 74,214 individuals across 13 non-UKB cohorts. These final exposure (33,181) and outcome (74,214) sample sizes represent the non-overlapping datasets used for our GSMR analyses.

By combining these GWAS datasets, we implemented GSMR to test the causal relationship between genetically predicted cortical morphology and intelligence, while minimizing bias due to population stratification, relatedness, or sample overlap.

3. Details of matSpD Determining Methods

The *matSpD* software uses matrix spectral decomposition to account for correlations among phenotypes or genetic markers in multiple testing corrections. By applying eigen decomposition to the correlation matrix, it estimates the effective number of independent variables (*te*) (10).

Instead of treating correlated phenotypes as fully independent, *matSpD* uses the variance explained by eigenvalues to determine how many variables contribute unique information. This effective number is then used to adjust the Bonferroni threshold, providing a less conservative correction while controlling the family-wise error rate.

In this study, we applied *matSpD* to cortical and subcortical correlation matrices to estimate *te*, which defined the Bonferroni-corrected significance threshold ($p < 0.05/te$).

Supplementary Results.

1. Bidirectional causal effects between cortical morphology and intelligence with different MR methods comparison

To avoid potential MR assumption violations, we complemented GSMR with additional MR methods. From Supplementary Figure 1, we observe overlapping significance across approaches in total surface area and the posterolateral temporal area, with forward MR results aligning across both absolute and relative analyses. (Table S2) For the dorsolateral prefrontal region, GSMR detected significance that was also supported by MR-IVW. Other sensitivity

methods did not reach significance for this region, which may reflect reduced power under these more conservative models.

Supplementary Figures.

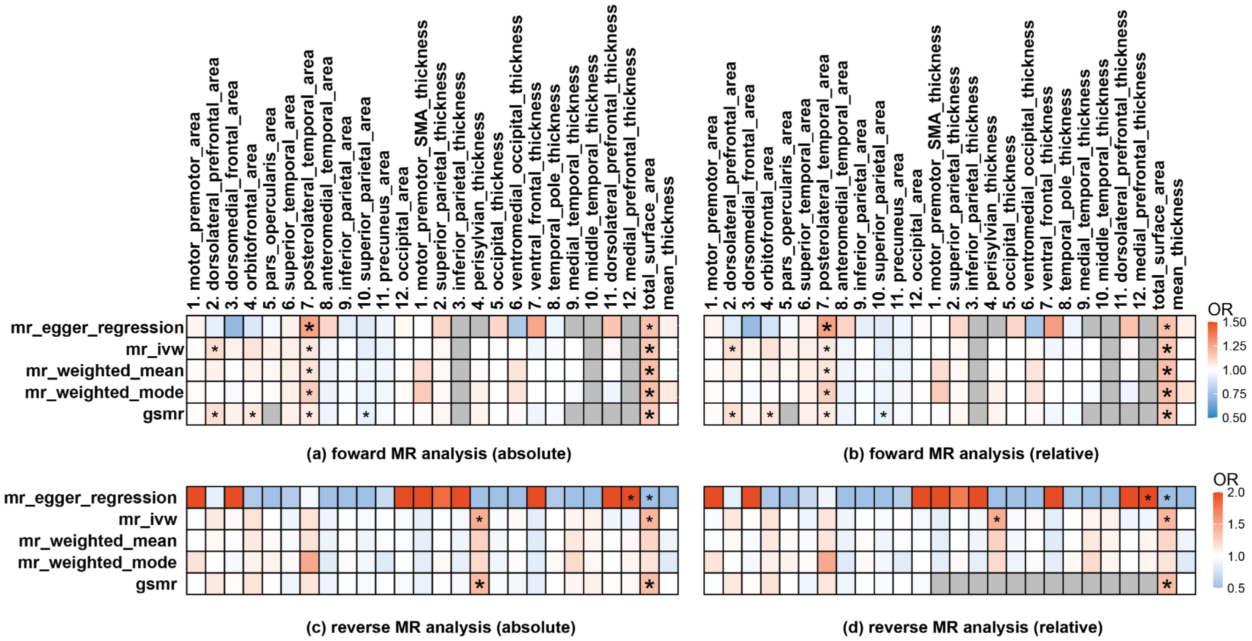

Figure S1. Bidirectional causal effects between cortical morphology and intelligence with different MR methods comparison. The heat maps display both (a)(b) forward and (c)(d) reverse Mendelian Randomization analyses. Big or small asterisks denote statistical ($p < 2.3e\text{-}3$, Bonferroni correction) or nominal ($p < 0.05$) significance. Gray boxes indicate missing data due to limitations such as restricted sample size or insufficient number of filtered single nucleotide polymorphisms (SNPs) meeting the inclusion threshold.

Supplementary Table.

Table S4. Instrument strength of genome-wide significant SNPs used in forward GSMR analyses across absolute cortical regions after pleiotropy filtering.

Forward GSMR IV (Significant Regions)								
Dorsolateral prefrontal area			Orbitofrontal area			Posterolateral temporal area		
IVs	F-stat	p	IVs	F-stat	p	IVs	F-stat	p
rs1015446	34.9401	3.40E-09	rs1015446	67.0930	2.59E-16	rs1023470	31.2423	2.28E-08
rs1078363	31.1565	2.38E-08	rs10206105	32.1853	1.40E-08	rs10809913	37.7563	8.02E-10
rs11020063	43.7425	3.75E-11	rs10942525	33.6054	6.75E-09	rs10830941	36.9991	1.18E-09
rs12676765	31.4399	2.06E-08	rs11579220	38.4984	5.48E-10	rs10878269	93.4330	4.20E-22
rs1456979	39.2943	3.64E-10	rs11767221	78.7286	7.13E-19	rs114921579	35.1844	3.00E-09
rs17235841	38.7386	4.85E-10	rs12626264	30.6217	3.14E-08	rs11669334	33.3128	7.85E-09
rs4868084	51.0033	9.22E-13	rs12724488	42.7068	6.36E-11	rs12372133	41.2045	1.37E-10
rs4924346	49.0254	2.53E-12	rs13233151	43.0543	5.32E-11	rs1637571	36.7538	1.34E-09
rs55743972	38.8828	4.50E-10	rs2282042	32.2479	1.36E-08	rs2166123	36.5849	1.46E-09
rs7093914	32.0145	1.53E-08	rs4681356	37.0855	1.13E-09	rs2991716	30.8946	2.72E-08
rs888812	63.4937	1.61E-15	rs7451690	33.6932	6.45E-09	rs305448	31.3495	2.16E-08
			rs9601373	31.3759	2.13E-08	rs3940842	55.6570	8.63E-14
						rs3999543	33.6925	6.46E-09
						rs61741508	49.1097	2.42E-12
						rs61909999	32.6133	1.12E-08
						rs669952	109.6043	1.20E-25
						rs68175985	130.1150	3.87E-30
						rs7154212	35.4162	2.66E-09
						rs7180955	30.5588	3.24E-08
						rs743029	30.9718	2.62E-08
						rs7560411	64.3856	1.02E-15
						rs7584720	43.4281	4.40E-11
						rs76014677	42.1546	8.43E-11
						rs79272390	38.5944	5.22E-10
						rs8025699	30.4096	3.50E-08
						rs8077219	38.0424	6.92E-10

Superior parietal area			Total surface area			
IVs	F-stat	p	IVs	F-stat	p	
rs10496078	44.1771	3.00E-11	rs10460587	46.6409	8.53E-12	
rs11175755	51.1470	8.57E-13	rs10496091	34.7754	3.70E-09	
rs11669334	63.1193	1.95E-15	rs11079849	36.9575	1.21E-09	
rs11979208	37.3260	9.99E-10	rs111379945	46.3455	9.91E-12	
rs12615194	38.4811	5.53E-10	rs11938781	41.1253	1.43E-10	
rs2033939	75.7102	3.28E-18	rs139590428	31.7002	1.80E-08	
rs231267	33.4160	7.44E-09	rs1951120	35.3004	2.83E-09	
rs4437022	31.4212	2.08E-08	rs1979477	30.0570	4.20E-08	
rs56111638	59.7040	1.10E-14	rs199534	226.5905	3.31E-51	
rs57205040	48.9207	2.67E-12	rs2764264	52.7473	3.79E-13	
rs62109937	29.9630	4.40E-08	rs28741121	31.0707	2.49E-08	
rs6858566	70.7845	3.98E-17	rs3786824	35.3381	2.77E-09	
rs73006822	125.2955	4.39E-29	rs4273712	121.2043	3.45E-28	
rs73578186	34.9155	3.44E-09	rs4685	51.4943	7.18E-13	
rs7563957	57.8581	2.82E-14	rs62283132	69.1649	9.06E-17	
rs888812	41.8035	1.01E-10	rs7220421	70.2833	5.14E-17	
rs9345124	33.0605	8.93E-09	rs76928645	83.5473	6.22E-20	
			rs7715167	33.0316	9.07E-09	
			rs77324770	36.3944	1.61E-09	
			rs8756	60.1247	8.90E-15	
			rs9266618	47.4516	5.64E-12	

Note: All listed SNPs exceed the conventional F > 10 threshold, indicating strong instruments. p-values reflect SNP–exposure associations from the discovery cortical GWAS.

Table S5. Instrument strength of genome-wide significant SNPs used in reverse GSMR analyses across absolute cortical regions after pleiotropy filtering.

Reverse GSMR IV (Significant Regions)						
Perisylvian thickness			Total surface area			
IVs	F-stat	p	IVs	F-stat	p	
rs12602130	29.7462	4.94E-08	rs12602130	29.7462	4.94E-08	
rs2251499	32.9820	9.31E-09	rs2251499	32.9820	9.31E-09	
rs2726491	34.3161	4.69E-09	rs2726491	34.3161	4.69E-09	
rs2920940	29.9647	4.39E-08	rs2920940	29.9647	4.39E-08	
rs4839715	33.1315	8.59E-09	rs4839715	33.1315	8.59E-09	
rs6023759	30.9693	2.62E-08	rs6023759	30.9693	2.62E-08	
rs7359397	30.9692	2.62E-08	rs7359397	30.9692	2.62E-08	
rs7468585	35.8442	2.14E-09	rs7468585	35.8442	2.14E-09	

Note: All listed SNPs exceed the conventional $F > 10$ threshold, indicating strong instruments. p-values reflect SNP–exposure associations from the discovery IQ GWAS.

Supplementary Reference.

1. Z. Zhu, *et al.*, Causal associations between risk factors and common diseases inferred from GWAS summary data. *Nat Commun* **9**, 224 (2018).

2. J. Bowden, G. Davey Smith, P. C. Haycock, S. Burgess, Consistent Estimation in Mendelian Randomization with Some Invalid Instruments Using a Weighted Median Estimator. *Genet Epidemiol* **40**, 304–314 (2016).

3. S. Burgess, C. N. Foley, E. Allara, J. R. Staley, J. M. M. Howson, A robust and efficient method for Mendelian randomization with hundreds of genetic variants. *Nat Commun* **11**, 376 (2020).

4. F. P. Hartwig, G. Davey Smith, J. Bowden, Robust inference in summary data Mendelian randomization via the zero modal pleiotropy assumption. *Int J Epidemiol* **46**, 1985–1998 (2017).

5. B. L. Pierce, H. Ahsan, T. J. Vanderweele, Power and instrument strength requirements for Mendelian randomization studies using multiple genetic variants. *Int J Epidemiol* **40**, 740–752 (2011).

6. J. Bowden, *et al.*, Improving the accuracy of two-sample summary-data Mendelian randomization: moving beyond the NOME assumption. *Int J Epidemiol* **48**, 728–742 (2019).

7. C. Makowski, *et al.*, Discovery of genomic loci of the human cerebral cortex using genetically informed brain atlases. *Science* **375**, 522–528 (2022).

8. C. Makowski, *et al.*, Larger cerebral cortex is genetically correlated with greater frontal area and dorsal thickness. *Proc Natl Acad Sci U S A* **120**, e2214834120 (2023).

9. J. E. Savage, *et al.*, Genome-wide association meta-analysis in 269,867 individuals identifies new genetic and functional links to intelligence. *Nat Genet* **50**, 912–919 (2018).

10. M.-X. Li, J. M. Y. Yeung, S. S. Cherny, P. C. Sham, Evaluating the effective numbers of independent tests and significant p-value thresholds in commercial genotyping arrays and public imputation reference datasets. *Hum. Genet.* **131**, 747–756 (2012).
